# A simple, clinically usable whole-body MRI system of joint assessment in adolescents and young people with juvenile idiopathic arthritis

**DOI:** 10.1093/rheumatology/keae117

**Published:** 2024-02-29

**Authors:** Varvara Choida, Timothy J P Bray, Niels van Vucht, Maaz Ali Abbasi, Alan P Bainbridge, Thomas Parry, Sue Mallett, Coziana Ciurtin, Margaret A Hall-Craggs

**Affiliations:** Centre for Medical Imaging, University College London, London, UK; Division of Medicine, Centre for Adolescent Rheumatology, University College London, UK; Department of Rheumatology, University College London Hospitals NHS Foundation Trust, London, UK; Centre for Medical Imaging, University College London, London, UK; Department of Imaging, University College London Hospitals NHS Foundation Trust, London, UK; Department of Imaging, University College London Hospitals NHS Foundation Trust, London, UK; Department of Imaging, University College London Hospitals NHS Foundation Trust, London, UK; Centre for Medical Imaging, University College London, London, UK; Department of Medical Physics, University College Hospitals Trust, London, UK; Centre for Medical Imaging, University College London, London, UK; Centre for Medical Imaging, University College London, London, UK; Division of Medicine, Centre for Adolescent Rheumatology, University College London, UK; Department of Rheumatology, University College London Hospitals NHS Foundation Trust, London, UK; Centre for Medical Imaging, University College London, London, UK; Department of Imaging, University College London Hospitals NHS Foundation Trust, London, UK

**Keywords:** whole-body, MRI, JIA, synovitis, activity, joint, Dixon, agreement, reliability, scoring

## Abstract

**Objectives:**

To introduce and evaluate a simple method for assessing joint inflammation and structural damage on whole-body MRI (WBMRI) in juvenile idiopathic arthritis (JIA), which is usable in clinical practice.

**Methods:**

The proposed system utilizes post-contrast Dixon WBMRI scans. Joints are assessed for synovitis (grade 0–2) and structural damage (present/absent) at 81 sites. The synovitis grading is based on features including above-normal intensity synovial enhancement, synovial hypertrophy, joint effusion, subarticular bone marrow oedema and peri-articular soft tissue oedema.

This system was evaluated in a prospective study of 60 young people (47 patients with JIA and 13 controls with non-inflammatory musculoskeletal pain) who underwent a WBMRI. Three readers (blinded to diagnosis) independently reviewed all images and re-reviewed 20 individual scans. The intra- and inter-reader overall agreement (OA) and the intra- and inter-reader Gwet’s agreement coefficients 2 (GAC2) were measured for the detection of a) participants with ≥1 joint with inflammation or structural damage and b) joint inflammation or structural damage for each joint.

**Results:**

The inter-reader OA for detecting patients with ≥1 joint with inflammation, defined as grade 2 synovitis (G2), and ≥1 joint with structural damage were 80% and 73%, respectively. The intra-reader OA for readers 1–3 was 80–90% and 75–90%, respectively. The inter-reader OA and GAC2 for joint inflammation (G2) at each joint were both ≥85% for all joints but were lower if grade 1 synovitis was included as positive.

**Conclusion:**

The intra- and inter-reader agreements of this WBMRI assessment system are adequate for assessing objective joint inflammation and damage in JIA.

Rheumatology key messages

- A clinically usable WBMRI-based assessment for joint inflammation and structural damage was developed for JIA
- Joint inflammation on WBMRI was detected with reliable intra- and inter-reader agreement
- WBMRI reporting times were conforming to clinical radiological practice for standard MRI scans

## Introduction

Whole-body MRI (WBMRI) enables the assessment of multiple joints, the entheses and axial skeleton for inflammation in one examination. This technique is promising for the monitoring of conditions like non-systemic juvenile idiopathic arthritis (JIA), which is heterogeneous and causes various patterns of joint inflammation, along with entheseal and axial inflammation in some JIA subtypes [1].

WBMRI has demonstrated its ability to detect joint inflammation in previous research studies [2]. However, there are several issues that need to be considered if WBMRI is to be a clinically useful tool. A WBMRI examination that is fit for purpose in JIA should image all the clinically important joints, be acceptable to patients, and be available and at reasonable cost. A framework for assessing and measuring joint inflammation and structural damage on whole-body scans is also required. This framework should provide a holistic objective assessment of musculoskeletal inflammation, be simple and reproducible between scan readers, and provide useful information for clinical decision-making.

Although there are a number of semi-quantitative MRI scoring systems for assessing disease activity in rheumatoid arthritis [3], psoriatic arthritis [4], spondyloarthritis [5, 6], and JIA [7–10], as well as proposed scoring systems for WBMRI [11, 12], these detailed systems are primarily designed for use in the research setting but are not practical for clinical care.

Therefore, our objectives were to develop a simple WBMRI-based joint assessment system that could be used in standard clinical care for patients with JIA, and to evaluate its intra- and inter-reader agreement.

## Methods

### Subjects

This was a prospective study, approved by London Queen Square Research Ethics Committee (15/LO/1475) and all participants provided informed written consent. The study complied with the ethical principles of the Declaration of Helsinki. We included 60 adolescent and young adult patients, under the care of the adolescent and young adult rheumatology team of University College London Hospital, with either JIA (*n* = 47) according to the International League of Associations for Rheumatology classification or musculoskeletal pain without inflammatory arthritis (controls, *n* = 13) according to the opinion of their treating rheumatologist. The exclusion criteria for both groups were any contraindications to undergo MRI scan or to receive gadolinium contrast. All participants underwent clinical examination before undergoing a WBMRI scan.

### Imaging acquisition

All MRI scans were performed on a 3-Tesla MRI system (Ingenia, Philips Healthcare). 3-D spoiled dual gradient echo Dixon sequences were obtained after administering 10 ml gadoteric acid meglumine (or 0.2 ml/kg if weight ≤50 kg). Coronal acquisitions of the whole body were divided in 6–8 anatomical stations, depending on the patient's height. Fat-water separation was performed in-line using vendor software, and four sets of images were produced: water-only, fat-only, in-phase and opposed-phase. The post-contrast stations 1–7 were ‘stitched’ into a whole-body image. The MRI parameters included TE: 1.31 & 1.9 ms, TR: 3.5 ms, flip angle: 10 degrees, acquisition matrix: 68–172 x 235–320 x 120 (depending on the station), voxel size 1.59–1.6 x 1.59–1.75 x 5 mm^3^, interslice gap: -2.5. The scan duration including the patient positioning, contrast administration and acquisition time of the post-contrast Dixon images was about 30 min.

Patients were positioned supine with their hands flat over the front of their thighs and the elbows close to their body. Two anterior phased array coils were placed over the trunk and lower limbs. A posterior coil was integrated in the scanner.

### Joint assessment on WBMRI

**Figure 1.**
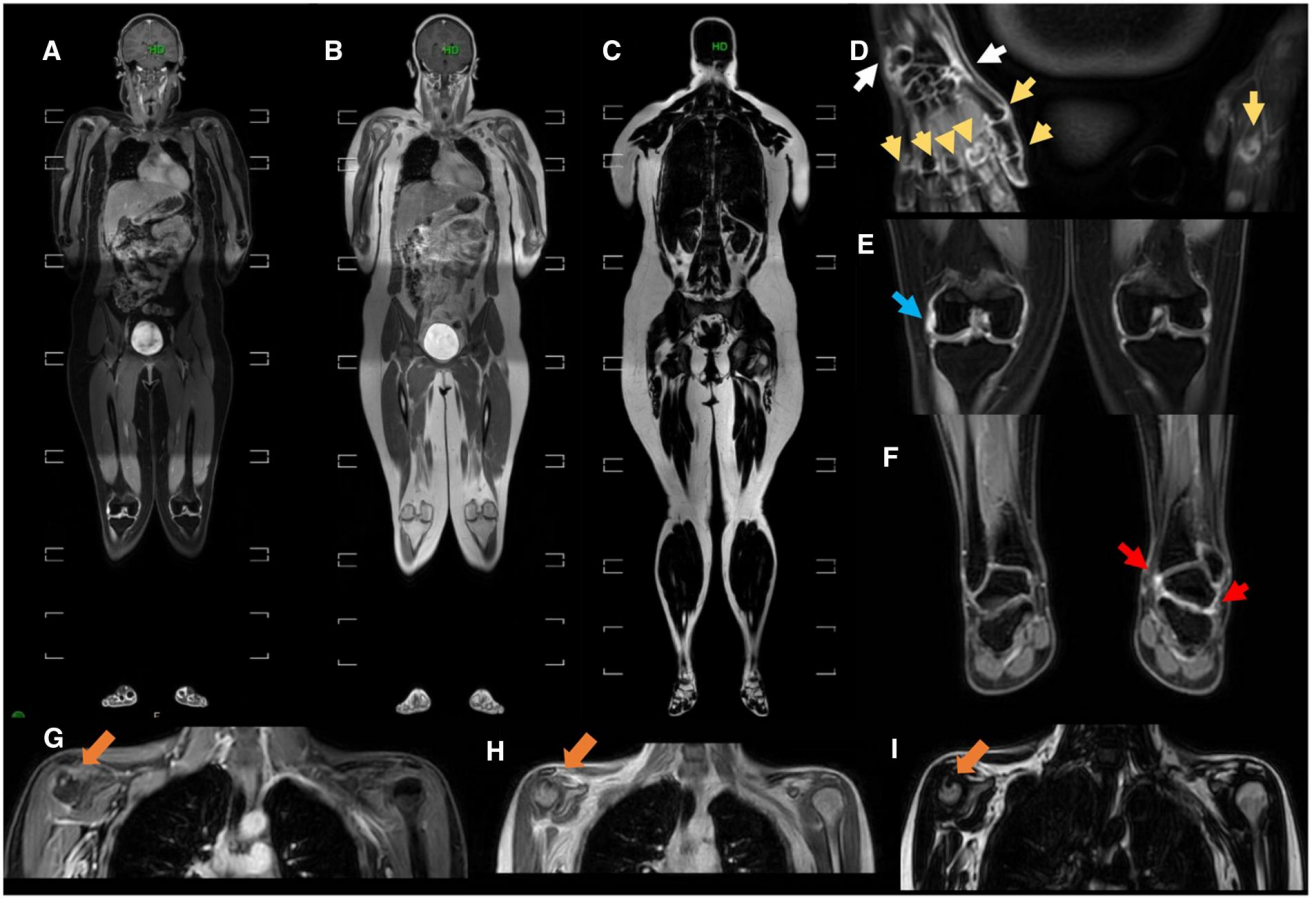
Post-contrast whole-body MRI images of two patients with polyarticular JIA (A-F and G-I). A. Whole-body water-only Dixon image. B. Whole-body in-phase Dixon image. C. Whole-body fat-only Dixon image. D. Grade 2 (G2) synovitis at the right wrist (white arrows), 1st–5th metacarpophalangeal joints and 1st interphalangeal joint and left 2nd metacarpophalangeal joints (yellow arrows). E. Right knee G2 synovitis (blue arrow). F. Left ankle and subtalar joint G2 synovitis (red arrows). G-I. Erosion (orange arrows) at right glenohumeral joint, demonstrating high signal on the water-only (G) and low signal on the in-phase (H) and fat-only images (I)

The peripheral joints, sacroiliac joints (SIJ) and cervical spine (CS) were assessed for inflammation and structural damage on WBMRI, according to the definitions described in Table 1. The joints assessed on WBMRI are listed in Table 2. Synovitis was graded as 0–2, whilst the SIJ and CS were assessed for inflammation dichotomously (Table 1). Grade 1 synovitis (G1S) was defined as above-normal intensity post-contrast synovial enhancement, whereas additional features were required for grade 2 synovitis (G2S). WBMRI images of joints with G2S and structural damage are shown in Fig. 1. The readers were instructed to use the multiplanar reconstruction facility on the Picture Archiving and Communication System (PACS) workstation as they would with normal scan reading practice. The readers had access to the full four sets of Dixon images and reviewed these in combination (Table 1).

**Table 1.** Definitions for whole-body MRI inflammation and structural damage at the joints and spine

**Joint synovitis (water-only Dixon images), grade 0-2** Grade 0: normal intensity post-contrast synovial enhancement. Grade 1: above-normal intensity synovial enhancement. Grade 2: above-normal intensity synovial enhancement with at least one of the following features: synovial hypertrophy, joint effusion, subarticular bone marrow oedema, or peri-articular soft tissue oedema. *Bone marrow oedema*: high signal within the bone marrow on post-contrast water-only Dixon images (low signal on fat-only and in-phase images).
**Sacroiliitis (water-only Dixon images), present/absent** Sacroiliitis was defined as the presence of subchondral or periarticular bone marrow oedema. Synovitis, enthesitis and capsulitis alone were not considered adequate to define sacroiliitis^a^ but were added in the comment section when present.
**Cervical spine inflammation (water-only Dixon images), present/absent** The cervical spine was assessed for anterior and posterior inflammation at each spinal level (C1-C7) and for synovitis in the atlantoaxial (AA) and atlantooccipital (AO) joints. The cervical spine was positive for inflammation if any of the above were positive. Anterior spinal inflammation: post-contrast enhancement at the vertebral corners or adjacent to the vertebral endplates. Posterior spinal inflammation: facet joint inflammation, or enthesitis of spinal ligaments.
**Peripheral joint structural damage (water-only, fat-only and in-phase images), present/absent** Defined as the presence of erosions or joint remodelling (typically seen at the hip and temporomandibular joints with flattening of the femoral head and condyle, respectively). *Erosions:* cortical defects with an intraosseous extension of signal abnormality, usually low signal on fat-only and in-phase images. Joint space narrowing could not be assessed reliably, so was not included; osteophytes were documented in the comment section when present.
**Chronic sacroiliitis (water-only, fat-only and in-phase images), present/absent** Defined as the presence of subarticular fat metaplasia, erosions, ankylosis or subchondral sclerosis^a^. *Fat metaplasia:* high signal within the bone marrow in fat-only and in-phase images, but low signal in water-only Dixon images. *Subchondral sclerosis* demonstrates low signal within bone marrow in in-phase images. *Ankylosis:* loss of signal from the iliac and sacral cortical bone with bone marrow signal crossing the joint space in fat-only and in-phase images.
**Structural damage at the cervical spine (water-only, fat-only and in-phase images)** Defined as the presence of erosions, fat metaplasia, ankylosis (bone bridges), new bone formation, or subluxation of AO, AA or subaxial joints.

a Modified definitions of Sieper *et al.* (*Ann Rheum Dis* 2009; 68; ii1-ii44) for Dixon imaging.

**Table 2.** Joints assessed on whole-body MRI and clinical examination per patient

Joints assessed clinically	Joints assessed on whole-body MRI
Temporomandibular joints (*n* = 2)	Temporomandibular joints (*n* = 2)
Shoulders (*n* = 2)	Shoulders (*n* = 2)
Sternoclavicular joints (*n* = 2)	Sternoclavicular joints (*n* = 2)
Acromioclavicular joints (*n* = 2)	Acromioclavicular joints (*n* = 2)
Elbows (*n* = 2)	Elbows (*n* = 2)
Wrists (*n* = 2)	Radiocarpal joints (*n* = 2), intercarpal joints (*n* = 2), radioulnar joints (*n* = 2), carpometacarpal joints (*n* = 10)
Metacarpophalangeal joints (*n* = 10)	Metacarpophalangeal joints (*n* = 10)
Hand interphalangeal joints (*n* = 28)	Hand interphalangeal joints (*n* = 28)
Hips (*n* = 2)	Hips (*n* = 2)
Knees (*n* = 2)	Knees (*n* = 2)
Ankles (*n* = 2)	Tibiotalar joints (*n* = 2)
Hindfoot joints (*n* = 2)	Posterior subtalar joints (*n* = 2), anterior/medial subtalar joints (*n* = 2), talonavicular joints (*n* = 2), calcaneocuboid joints (*n* = 2)
Midfoot joints (*n* = 2)	Cuneonavicular joints (*n* = 2), cuboideonavicular joints (*n* = 2), cuneocuboid joints (*n* = 2), intercuneiform joints (*n* = 2), tarsometatarsal joints (*n* = 10)
Metatarsophalangeal joints (*n* = 10)	Metatarsophalangeal joints (*n* = 10)
Foot interphalangeal joints (*n* = 28)	Foot interphalangeal joints (*n* = 28)
Axial joints	
Sacroiliac joints (*n* = 2)	Sacroiliac joints (*n* = 2)
Cervical spine (*n* = 1)	C1-C7
**Total: 81 joints**	**Total: 116 joints + cervical spine**

### Combining joints post-image review according to clinical assessment

After the image review, we combined the joints assessed on WBMRI into 81 joints to harmonize with the clinical assessment of patients with JIA (Table 2). We grouped the small joints of the wrist and foot (which were graded for synovitis individually) onto the wrist, hindfoot and midfoot joints and assigned the highest synovitis grading of the small joints to these complex joints.

### Reading sessions

Three musculoskeletal radiologists (MAA, NvV and MHC) with 9, 5, over 25 years of experience respectively reviewed the post-contrast WBMRI images of 60 patients independently, blinded to the diagnosis (JIA or controls) and clinical information in random order. The second round of reading sessions started five weeks after the completion of the first round of WBMRI assessments. All radiologists re-reviewed a subset of 20 WBMRI scans; 17 and 3 scans of JIA patients and controls, respectively, were selected randomly. During each reading session, an observer (VC) documented the reader’s findings in a schematic scoring form (Supplementary Fig. 1, available at *Rheumatology* online) and recorded the reporting time. Joints that could not be assessed were crossed on the form.

### Training before the reading sessions

Two training sessions were organized for the musculoskeletal radiologists, including hands-on training on WBMRI assessment, using a training set of images. The senior radiologist MHC (experienced in reading JIA whole-body scans) was the trainer at both sessions. The radiologists were given a handbook with the scoring methodology.

### Statistical analysis

We measured the intra- and inter-reader agreement and reliability in three levels on the following outcomes.

1. The detection of ≥ 1 joint per participant with a) joint inflammation (without including G1S), b) joint inflammation (including G1S), c) structural damage.
2. The total inflammation score (sum of joint inflammation/patient, with and without G1S; 0-162) and total structural damage score/patient (0-81).
3. The detection of a) joint inflammation (without including G1S), b) joint inflammation (including G1S) and c) structural damage at the same joint. The agreement was calculated per joint, e.g. wrist based on the assessment of all wrist joints on WBMRI. The metacarpophalangeal joints and finger interphalangeal joints (IPJ) were grouped together as hand joints and the agreement was measured collectively for all hand joints. The same approach was applied to the metatarsophalangeal joints and foot IPJ which were grouped as forefoot joints.

At levels 1 and 3, we measured the overall agreement (OA), positive specific agreement (PA), and negative specific agreement (NA) between the readers, as described previously [13] (Supplementary Table S1, available at *Rheumatology* online). In addition, we measured the intra- and inter-reader reliability by Gwet’s agreement coefficient 2 [GAC2, (2014)]. The GAC2 was interpreted as: poor (below 0), slight (0–0.20), fair (0.21–0.40), moderate (0.41–0.60), substantial (0.61–0.80), or almost perfect (0.81–1.00) [14]. At level 2, the inter-reader/intra-reader reliability was estimated by the two-way random/mixed effects (respectively), single rater, absolute agreement form of intraclass correlation coefficient (ICC). According to ICC, the reliability was graded as poor (<0.5), moderate (0.5–0.75), good (0.75–0.9), and excellent (>0.90) [15].

Joints that could not be assessed by all radiologists were excluded. The statistical analysis was performed using STATA/MP2 version 16.

## Results

### Participants

Forty-seven (29 female, 18 male) patients with JIA and 13 (11 female, 2 male) controls underwent a WBMRI scan. The median age was 18 years (range 14–24) for people with JIA and 16 years (range 15–19) for controls. On examination, 25/47 (53%) patients with JIA had either ≥1 active joint and/or clinical sacroiliitis and the remaining patients had neither. All differentiated JIA subtypes were represented; the frequency of each subtype, patients’ treatments and disease activity measures are summarized in Supplementary Table S2, available at *Rheumatology* online.

### Assessable joints and reporting time

237/4860 (4.9%) joints could not be assessed by all readers. The reasons by decreasing frequency were: 1) joints not included fully in the field of view due to patient’s positioning and dimensions [11 (9%) elbow joints, 211 (13%) forefoot joints] or omitted by error (SIJs in one patient), 2) metal artefacts due to dental braces (six TMJs) or orthopaedic surgery (two wrists, one ankle) 3) joint replacement (three hip joints) and 4) bright artefact (one knee). At the second scoring, no additional joints were identified as not assessable.

The median reporting time (interquartile range) in min at the first round was 14 (10.5–19.5) for reader 1; 9.5 (8–14) for reader 2; and 7 (5.5–8.5) for reader 3, who was most experienced in reading WBMRI scans of patients with JIA.

### Frequency of synovitis and structural damage detection by readers and participant group

Joint inflammation (defined as G2S in peripheral joints) was detected in 244 (6.8%), 211 (5.9%) and 277 (7.7%) joints of participants with JIA and in 6 (0.6%), 0 and 3 (0.3%) joints of controls, by reader 1, 2 and 3 respectively. Joint inflammation (including G1S) was detected in 429 (11.9%), 361 (10%) and 333 (9.2%) joints in participants with JIA and in 33 (3.2%), 17 (1.7%) and 7 (0.7%) joints in controls, by reader 1, 2 and 3 respectively.

Structural damage was detected in 61 (1.7%), 53 (1.5%) and 82 (2.3%) joints in participants with JIA and in 3 (0.3%), 4 (0.4%) and 3 (0.3%) joints in controls, by reader 1, 2 and 3 respectively.

### Inter-reader agreement for the detection of participants with at least one joint with inflammation/structural damage on WBMRI

If we defined peripheral joint inflammation as G2S, the inter-reader OA for the detection of a participant with ≥1 joint with inflammation was 80% (95% CI 74, 85) between the readers. The PA was 82% (95% CI 77, 87), which means that if a reader scores ≥1 joint with inflammation in a patient, there is an 82% probability that another reader will score ≥1 joint with inflammation in the same patient. The NA was 77% (95% CI 70, 83), which indicates that if a reader does not identify any joints with inflammation in a patient, there is a 77% probability that another reader will not identify any. The inter-reader reliability for detecting a participant with joint inflammation was substantial [GAC2 = 61% (95% CI 44, 77)]. If we defined peripheral joint inflammation as G1S or G2S, the inter-reader OA for detecting a participant with ≥1 joint with inflammation was 78% (95% CI 71, 83), PA was 86% (95% CI 82, 90), NA was 44% (95% CI 34, 56) and GAC2 was consistent with substantial reliability [69% (95% CI 54, 84)].

In terms of structural damage, the OA for detecting a participant with ≥1 joint with structural damage was 73% (95% CI 66, 79), PA was 72% (95% CI 65, 78), NA was 75% (95% CI 68, 80) and GAC2 was consistent with moderate reliability [47% (95% CI 30, 64)].

### Inter-reader agreement for the detection of the same joint with inflammation or structural damage

**Figure 2.**
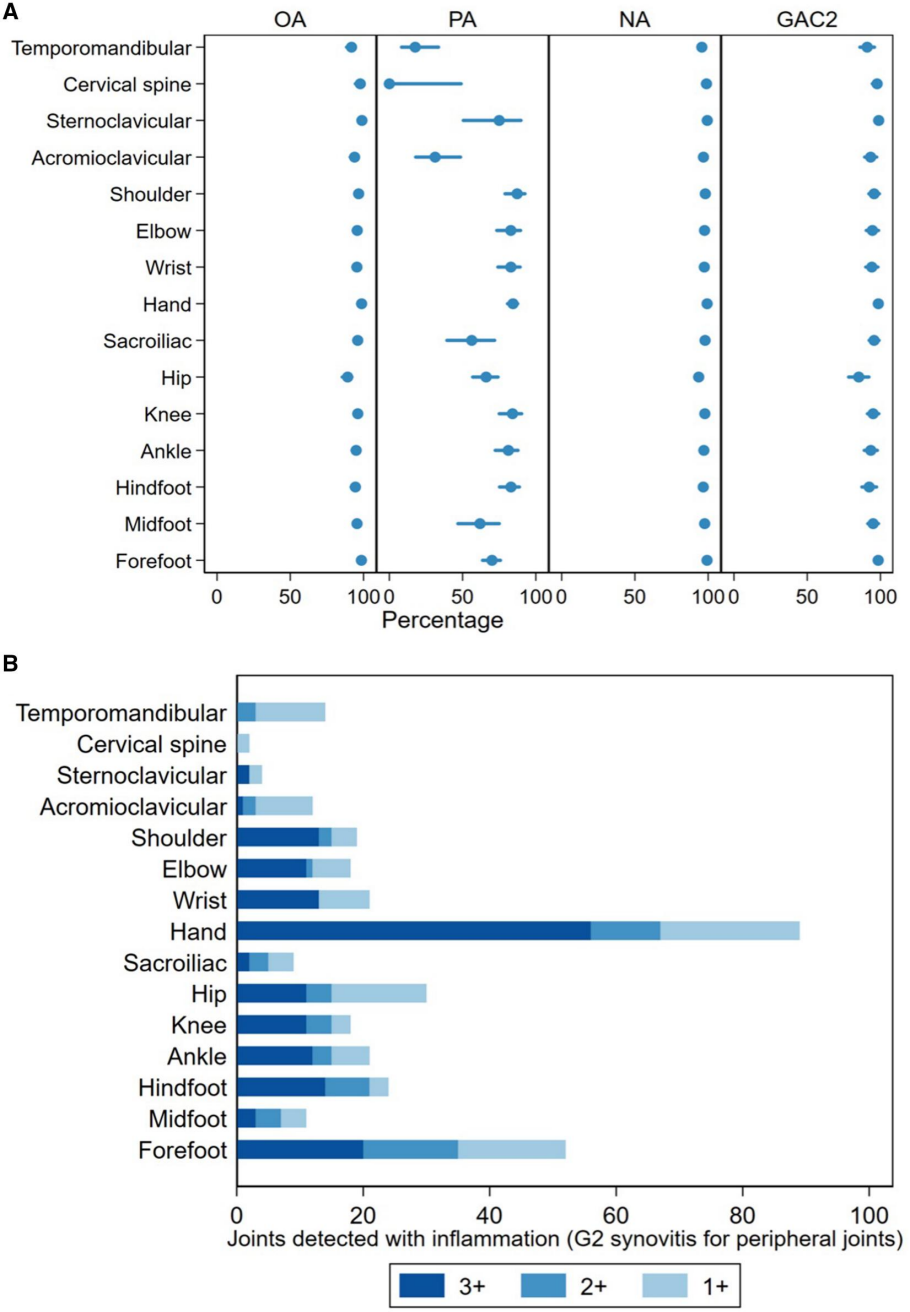
Inter-reader agreement on joint inflammation detected on WBMRI scans by 3 independent readers, per joint. A. Inter-reader OA, PA, NA, GAC2 (%) agreement for joint inflammation, defined as G2 synovitis in peripheral joints. The bars indicate the 95% confidence interval. B. The graph shows the number of joints scored positive for inflammation (G2 synovitis for peripheral joints) by three (3+), two (2+) and one (1+) reader(s) in different colours. Sixty patients (47 patients with JIA and 13 controls) were included. G2: grade 2, GAC2: Gwet’s agreement coefficient 2, NA: negative agreement; OA: overall agreement; PA: positive agreement; WBMRI: whole-body MRI

The inter-reader OA, PA, NA and GAC2 for joint inflammation (without including G1S) are presented in Fig. 2A and Supplementary Table S3, available at *Rheumatology* online. The number of joints with inflammation (not including G1S) according to one, two or all readers per joint is displayed in Fig. 2B. The inter-reader PA for joint inflammation was 0 for the CS as only two patients with JIA were identified with CS inflammation by reader 1 and 3 respectively.

Overall, the OA, PA and NA were lower if G1S was included in the definition of peripheral joint inflammation (Supplementary Table S4, available at *Rheumatology* online). The number of joints detected with inflammation (including G1S) by one, two or all readers per joint are shown in Supplementary Fig. S2, available at *Rheumatology* online.

The OA, NA and GAC2 on structural damage were above 90% except for the SIJ (OA : 84%, GAC2:78%, Supplementary Table S5, available at *Rheumatology* online). The PA for structural damage was low (0–31%) in the small joints of the wrist, hand, forefoot, midfoot, hindfoot and higher in the larger joints [glenohumeral (76%), knee (75%), sacroiliac (51%), elbow (50%), ankle (50%)], except for the hip joint (31%).

### Intra-reader agreement and reliability for the detection of participants with at least one joint with inflammation or structural damage on WBMRI

The intra-reader agreement and reliability for readers 1, 2 and 3 for the above are displayed in Table 3.

**Table 3.** The intra-reader agreement of three readers for detecting patients with joint inflammation or structural damage

	Reader	OA	PA	NA	GAC2
Joint inflammation (G2 synovitis for peripheral joints)	1	80 (58, 92)	82 (61, 93)	78 (55, 91)	60 (22, 99)
	2	90 (70, 97)	89 (67, 97)	91 (72, 97)	80 (52, 100)
	3	90 (70, 97)	90 (70, 97)	90 (70, 97)	80 (51, 100)
Joint inflammation (G1 or G2 synovitis for peripheral joints)	1	95 (76, 99)	97 (86, 100)	67 (21, 94)	94 (81, 100)
	2	75 (53, 89)	81 (63, 92)	62 (36, 82)	55 (14, 96)
	3	80 (58, 92)	86 (69, 94)	67 (39, 86)	66 (29, 100)
Structural damage	1	90 (70, 97)	90 (70, 97)	90 (70, 97)	80 (51, 100)
	2	80 (58, 92)	78 (55, 91)	82 (61, 93)	60 (22, 99)
	3	75 (53, 89)	76 (55, 89)	74 (51, 88)	50 (9, 92)

The joints of 20 patients (17 patients with JIA and 3 controls) were assessed twice for joint inflammation and structural damage on whole-body MRI by 3 readers. The intra-reader agreement on detecting ≥ 1 joint with inflammation/structural damage was calculated for each reader. GAC2: Gwet’s agreement coefficient 2, G1: grade 2, G1: grade 1, NA: negative specific agreement, OA: overall agreement, PA: positive specific agreement. Data are % (95% CI).

### Intra-reader agreement for joint inflammation and structural damage at the same joint

**Figure 3.**
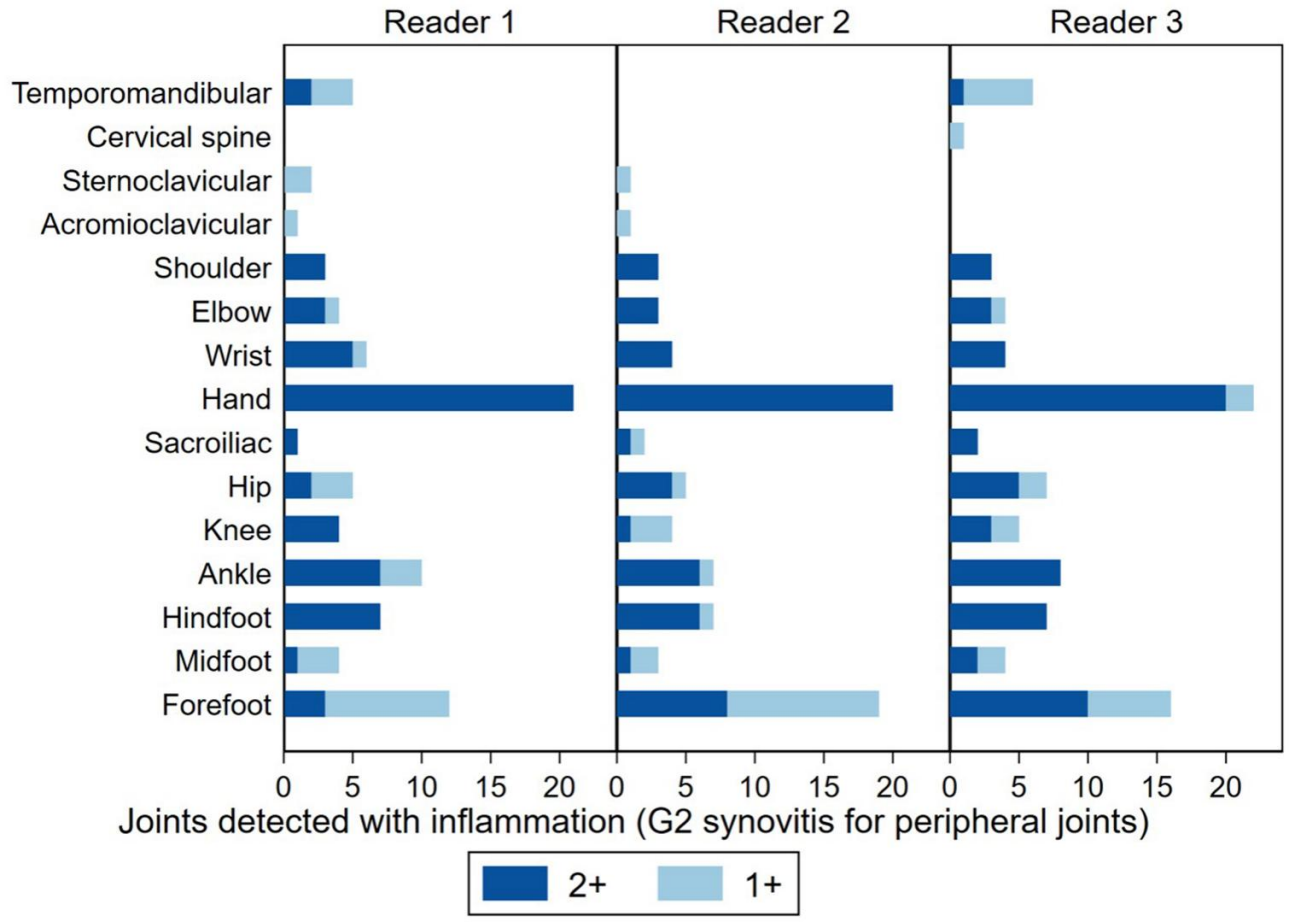
Intra-reader agreement on the joints detected with inflammation (G2 synovitis) for 3 readers, per joint. A. Reader 1; B. Reader 2; C. Reader 3. The whole-body MRI scans of 20 patients (17 patients with JIA, 3 controls) were assessed twice by each reader. The graph shows the number of joints positive for inflammation (G2 synovitis for peripheral joints) detected by the same reader once (1+) or twice (2+) in all patients for each joint on the *y*-axis. G2: grade 2

The number of joints detected with inflammation (without including G1S) in one and both reading sessions by readers 1–3 are displayed in Fig. 3. The number of joints detected with inflammation (including G1S), or structural damage, in one and both reading sessions by the readers are displayed in Supplementary Fig. S3, available at *Rheumatology* online. The respective intra-reader OA, PA, NA and GAC2 are not shown as the number of positive joints with joint inflammation or structural damage in this subset of patients was low for many of the joints.

### Intra- and inter-reader reliability on total inflammation and structural damage scores per patient

The intra- and inter-reader reliability for total inflammation scores (with and without G1S) were excellent. The intra- and inter-reader reliability for total structural damage scores were moderate to good. The ICCs are summarized in Supplementary Tables S6 and S7, available at *Rheumatology* online.

## Discussion

In this study, we introduced and evaluated a simple joint assessment system for patients with JIA based on post-contrast WBMRI Dixon images. The joint assessment on WBMRI covered all the joints that are assessed in patients with JIA in standard clinical care, including the SIJ, providing a comprehensive assessment of disease activity and structural damage. The detection of a patient with joint inflammation by one reader was associated with a high probability that another reader, or the same reader at a second reading, will identify the same patient as having joint inflammation on WBMRI. We selected a cut-off of one joint with inflammation to assess the intra- and inter-reader agreement at the patient level as the detection of one active joint on clinical assessment is likely to influence the treatment plan.

At the joint level, the inter-reader reliability for joint inflammation, defined as G2S, was almost perfect for all joints. The positive agreement was high for many of the frequently involved joints in JIA, such as the knee and ankle joints. This suggests that WBMRI-detected joint inflammation is a potential imaging biomarker of JIA inflammation as it can be measured reliably at multiple joints.

Defining joint inflammation in peripheral joints as G2S, without including G1S, was associated with a higher inter-reader agreement. Our description of G1S is not classified as synovitis based on the OMERACT definitions because of the absence of synovial hypertrophy [3, 4, 16]. However, given the qualitative assessment of synovial hypertrophyon WBMRI, G1S could be used to identify the intermediate cases between definite synovitis and normal synovium, despite its uncertain clinical significance. In the clinical setting, G1S should not be treated as definite synovitis.

Structural damage was detected rarely on WBMRI. The inter-reader agreement was higher for the detection of structural damage in large joints compared with small joints (except for hip joints) as the more limited spatial resolution of WBMRI interferes with the assessment for erosions in small joints. Hip structural damage is more complex to define on MRI due to the various structural changes reported in JIA [17], including growth disturbances [18]. Cartilage loss cannot be appreciated adequately on WBMRI.

The TMJ was characterized by low inter-reader agreement on joint inflammation and structural damage. To improve the reliability of the TMJ assessment, a higher spatial resolution scan is likely required. We chose not to apply a head coil to patients to make the examination more comfortable. In addition, training for musculoskeletal radiologists in the assessment of TMJ might be needed due to their relatively limited experience with TMJ imaging and the multiple components of structural damage in this joint [19].

The SIJ was the most frequently detected joint with structural damage. However, the inter-reader PA was modest for structural damage and inflammation. Dedicated images of the SIJs, in addition to the WBMRI protocol, may be needed to improve the inter-reader agreement and offer a more detailed evaluation of these joints which is useful for detecting disease progression in patients with juvenile spondyloarthritis. Our protocol is based on Dixon imaging, which is a reliable sequence to detect bone marrow oedema and fat metaplasia in the SIJ of patients with spondyloarthritis [20, 21].

Post-contrast Dixon sequences have many additional benefits over the other sequences proposed for the use of WBMRI in inflammatory arthritis [11, 12]. Firstly, the water-only Dixon images display a better signal-to-noise ratio and a more uniform fat suppression than short-tau inversion recovery (STIR) [22, 23]. Secondly, compared with STIR, the Dixon technique can be combined with contrast administration. Contrast-enhanced MRI improves the assessment for synovitis [24, 25] and is recommended for the joint assessment in JIA [26]. The development of more sensitive non-contrast MRI imaging techniques for synovitis is desirable as these would be more ‘patient-friendly’. Thirdly, the T1-weighted in-phase post-contrast images can substitute the need for pre-contrast T1-weighted images, which means inflammation and structural damage can be assessed with one sequence as previously shown [27]. The use of Dixon gradient echo sequences makes this WBMRI protocol faster than other described protocols [12, 28, 29]. Although prolonged post-contrast imaging acquisition can potentially lead to false-positive synovitis detection [30], this pattern was not observed in the control group (G2S not seen specifically in joints imaged late *vs* early).

Moreover, the simpler methodology of the proposed joint assessment system and the schematic reporting of joint pathology in a scoring form resulted in very modest reading times (median reporting time per scan: 7–14 min) comparable to the reporting times of MRI scans in clinical practice [31]. However, readers were not asked to report incidental findings which can prolong reporting times.

Other strengths of our study were the recruitment of a relatively large number of patients, given that the prevalence of JIA in the UK is 1 in 1000 [32] (10 times less frequent than rheumatoid arthritis [33]), and the inclusion of all JIA subtypes with different patterns of joint inflammation. Moreover, we included a control group and blinded the reading process to decrease bias. In addition, the low detection rate of G2S in this group supports the validity of the WBMRI-detected joint inflammation.

On the other hand, a limitation of our study is that it involved patients and readers from one tertiary centre. We did not include younger children; therefore, we did not assess our methodology in patients at earlier stages of their skeletal development and disease. As non-specific bone marrow changes and joint effusions are described in healthy children [34], it would be important to assess the specificity of WBMRI assessment in younger ages. In addition, we devised new criteria for the definition of joint inflammation by encompassing additional components, namely osteitis, joint effusion and peri-articular soft tissue inflammation. This definition was not developed after consultation with other experts (consensus). However, these features are already included in other joint assessment systems, albeit reported individually [8, 35–38]. Finally, a limitation was that joint inflammation and structural damage were encountered in a small proportion of joints. This is expected given the large number of joints assessed on WBMRI compared with the much lower frequency of inflamed joints in patients with active disease, but also due to the inclusion of inactive patients in our study. We addressed this by measuring the PA as well as the OA and NA. The GAC2 was selected over Cohen’s kappa statistic, as the former is less affected by the Cohen’s kappa paradox [39]. Correlation with clinical findings and patients’ acceptability of WBMRI have not been assessed here as they will be reported separately.

The role of WBMRI in monitoring joint inflammation and supporting treatment decisions for patients with JIA should be investigated in future research studies. Our proposed WBMRI protocol and joint assessment methodology provide a framework to measure joint inflammation and structural damage. Its ability to detect disease progression or change in disease activity requires further assessment in a longitudinal study. We anticipate that this joint assessment system would be easy to understand by rheumatologists as it mirrors their clinical assessment. With the development of the relevant software, the disease activity on WBMRI could be presented to rheumatologists and patients in one image, for example by using a colour-coded whole-body image, which would enhance their understanding of the findings. Finally, this WBMRI assessment system has real potential for clinical translation as the scanning and reporting times, which could be reduced more in the future with the application of deep learning tools, are in line with other protocols used in clinical practice.

## Conclusion

We developed a joint assessment system for evaluating joint inflammation and structural damage in patients with JIA based on a Dixon-based WBMRI after contrast administration. In a prospective study, we demonstrated that the assessment of multiple sites was feasible and time-efficient in terms of scanning and reporting times. The intra- and inter-reader agreements were satisfactory for joint inflammation but more uncertain for structural damage as it was detected rarely. Overall, this system provides a sufficient agreement between readers for its use in the assessment of patients with JIA. Future research studies can refine and use this system to investigate the potential clinical benefits of measuring the disease activity by WBMRI.

## Supplementary Material

keae117_Supplementary_Data

## Data Availability

The data underlying this article will be shared on reasonable request to the corresponding author.
