# Supplementary material for: A simple, clinically usable whole-body MRI system of joint assessment in adolescents and young people with juvenile idiopathic arthritis
Source: Rheumatology (Oxford). 2024 Feb 29;63(SI2):SI219–27. doi: 10.1093/rheumatology/keae117 (PMC11381680; doi:10.1093/rheumatology/keae117)
Supplement: keae117_Supplementary_Data [file keae117_supplementary_data.docx]

**SUPPLEMENTARY MATERIAL**

Title: **A simple, clinically usable whole-body MRI system of joint assessment in adolescents and young people with juvenile idiopathic arthritis**

**Authors: Varvara Choida^1,2,3^, Timothy J.P. Bray^1,4^, Niels van Vucht^4^, Maaz Ali Abbasi^4^**, **Alan P. Bainbridge^1,5^, Thomas Parry^1^, Sue Mallett^1^, Coziana Ciurtin^2,3^, Margaret A. Hall-Craggs^1,4^**

**Affiliations**
1. Centre for Medical Imaging, University College London, London, UK.

2. Centre for Adolescent Rheumatology, Division of Medicine, University College London, UK

3. Department of Rheumatology, University College London Hospitals NHS Foundation Trust, London, UK

4. Department of Imaging, University College London Hospitals NHS Foundation Trust, London, UK

5. Department of Medical Physics, University College Hospitals Trust, London, UK

Correspondence to: Professor Margaret Hall-Craggs, Centre for Medical Imaging, University College London, 2nd Floor Charles Bell House, 43-45 Foley Street, W1W 7TS London, UK. Email: m.hall-craggs@ucl.ac.uk


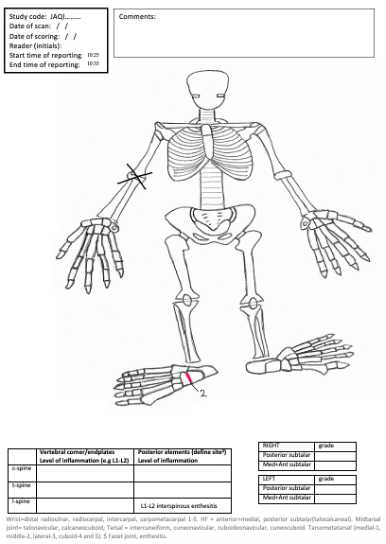


**Supplementary Figure S1.** Example of the recording of whole-body MRI findings on the scoring form for each participant. Right elbow crossed as not assessable, right talonavicular joint marked with red colour and 2 recorded next to the joint to indicate grade 2 synovitis.

**Supplementary Table S1.** Statistical analysis for inter- and intra-reader agreement

| **Inter-reader agreement** | | | |
| --- | --- | --- | --- |
|  | | **Reader 2** | |
|  |  | **+** | **-** |
| **Reader 1** | **+** | a | b |
|  | **-** | c | d |
|  | | | |
|  | | **Reader 3** | |
|  |  | **+** | **-** |
| **Reader 1** | **+** | e | f |
|  | **-** | g | h |
|  | | | |
|  | | **Reader 3** | |
|  |  | **+** | **-** |
| **Reader 2** | **+** | i | j |
|  | **-** | k | l |
| OA = (a+e+i+d+h+l)/(a to l), PA = ((a+e+i)*2)/((a+e+i)*2)+(b+c+f+g+j+k)),  NA = ((d+h+l)*2)/((d+h+l)*2)+(b+c+f+g+j+k)) | | | |
| **Intra-reader agreement (for each reader 1-3)** | | | |
|  | | **Reading 2** | |
|  |  | **+** | **-** |
| **Reading 1** | **+** | m | n |
|  | **-** | o | p |
| OA= m+d/m+n+o+p, PA= 2*m/2*m + (n+o), NA= 2*p/2*p+(n+o) | | | |
| +: positive for joint inflammation/structural damage on WBMRI, -: negative for joint inflammation/structural damage on WBMRI, a-p: number of joints/patients depending on analysis, NA: negative specific agreement, OA: overall agreement, PA: positive specific agreement, WBMRI: whole-body MRI. The Wilson 95% confidence intervals were calculated. | | | |

Supplementary Table S2 Frequency of JIA subtypes , treatments, and disease activity measures in 47 patients with JIA

| **JIA subtypes, n (%)** | |
| --- | --- |
| Enthesitis-related arthritis | 13 (28) |
| Extended oligoarticular | 12 (26) |
| Polyarticular RF-negative | 9 (19) |
| Polyarticular RF-positive | 4 (9) |
| Psoriatic | 4 (9) |
| Systemic-onset | 3 (6) |
| Persistent oligoarticular | 2 (4) |
| **Treatments, n (%)** | |
| No DMARD, | 8 (17) |
| Conventional synthetic DMARD (≥1) | 12 (26) |
| Biologic DMARD monotherapy | 10 (21) |
| Combination of biologic and conventional synthetic DMARD, | 17 (36) |
| **Disease activity measures, median (interquartile range)** | |
| Active joint count | 0 (0-4) |
| Limited joint count | 2 (0-5) |
| PhGA (0-10)* | 2 (0-3) |
| PtGA (0-100 mm)* | 25 (5-65) |
| JADAS10-CRP | 6.74 (1.50-13.00) |
| CHAQ (0-3), n=46 | 0.50 (0-1.38) |
| Erythrocyte sediment ratio (mm/hr), n=45 | 6 (2-8) |
| CRP (mg/L) | 1.1 (0.6- 2.9) |
| **Clinical sacroiliitis, n (%)** | 6 (13) |
| CHAQ: Childhood Health Assessment Questionnaire, CRP: C-reactive protein, DMARD: disease-modifying anti-rheumatic drug, JADAS10-CRP: Juvenile Arthritis Disease Activity Score (maximum 10 active joints)-CRP, PhGA: physician’s global assessment of disease activity, PtGA: patient’s global assessment of well-being, RF: rheumatoid factor*Scale from low to high severity. | |

**Supplementary Table S3.** The inter-reader OA, PA, NA, and GAC2 estimates for the identification of the same joint with inflammation (G2 synovitis for peripheral joints)

| **Joints** | **OA** | **PA** | **NA** | **GAC2** |
| --- | --- | --- | --- | --- |
| Temporomandibular | 92 (88, 94) | 18 (8, 34) | 96 (94, 97) | 91 (86, 96) |
| Cervical spine | 98 (94, 99) | 0 (0, 49) | 99 (97, 100) | 98 (94, 100) |
| Sternoclavicular | 99 (97, 100) | 75 (51, 90) | 99 (99, 100) | 99 (97, 100) |
| Acromioclavicular | 94 (91, 96) | 31 (18, 49) | 97 (95, 98) | 93 (89, 97) |
| Glenohumeral | 97 (94, 98) | 87 (79, 93) | 98 (97, 99) | 96 (92, 99) |
| Elbow | 96 (93, 97) | 83 (73, 90) | 98 (96, 99) | 95 (90, 99) |
| Wrist | 95 (93, 97 | 83 (74, 89) | 97 (96, 98) | 94 (90, 98) |
| Hand | 99 (98, 99) | 84 (81, 88) | 99 (99, 99) | 99 (98, 99) |
| Sacroiliac | 96 (93, 98) | 56 (39, 72) | 98 (97, 99) | 96 (92, 99) |
| Hip | 89 (85, 92) | 66 (57, 74) | 94 (91, 95) | 85 (78, 92) |
| Knee | 96 (94, 98) | 84 (75, 90) | 98 (96, 99) | 95 (91, 99) |
| Ankle | 95 (92, 97) | 81 (72, 88) | 97 (95, 98) | 93 (89, 98) |
| Hindfoot | 94 (92, 96) | 83 (75, 89) | 97 (95, 98) | 92 (87, 97) |
| Midfoot | 96 (93, 97) | 62 (47, 75) | 98 (96, 99) | 95 (91, 99) |
| Forefoot | 99 (98, 99) | 70 (64, 76) | 99 (99, 99) | 98 (98, 99) |
| The joints of 60 patients (47 patients with JIA and 13 controls) were assessed independently for joint inflammation on whole-body MRI scans by 3 readers. GAC2: Gwet’s agreement coefficient 2, G2: grade 2 synovitis, NA: negative specific agreement, OA: overall agreement, PA: positive specific agreement. Data are % (95% CI). | | | | |

**Supplementary Table S4.** The inter-reader OA, PA, NA, and GAC2 estimates for the identification of the same joint with joint inflammation (G1 synovitis included)

| **Joints** | **OA** | **PA** | **NA** | **GAC2** |
| --- | --- | --- | --- | --- |
| Temporomandibular | 84 (80, 88) | 51 (42, 60) | 91 (88, 93) | 78 (70, 87) |
| Cervical spine | 98 (94, 99) | 0 (0, 49) | 99 (97, 100) | 98 (94, 100) |
| Sternoclavicular | 91 (88, 94) | 33 (22, 47) | 95 (93, 97) | 90 (85, 95) |
| Acromioclavicular | 78 (74, 82) | 43 (35, 52) | 87 (84, 89) | 69 (58, 79) |
| Glenohumeral | 82 (77, 85) | 62 (54, 69) | 88 (85, 90) | 71 (61, 81) |
| Elbow | 82 (77, 85) | 63 (55, 70) | 88 (85, 90) | 71 (60, 81) |
| Wrist | 85 (81, 89) | 67 (59, 74) | 91 (88, 93) | 78 (69, 86) |
| Hand | 98 (98, 99) | 82 (78, 85) | 99 (99, 99) | 98 (98, 99) |
| Sacroiliac | 96 (93, 98) | 56 (39, 72) | 98 (97, 99) | 96 (92, 99) |
| Hip | 84 (80, 88) | 71 (65, 77) | 89 (86, 91) | 73 (63, 83) |
| Knee | 90 (87, 93) | 71 (62, 79) | 94 (92, 96) | 87 (80, 93) |
| Ankle | 85 (81, 89) | 73 (66, 79) | 90 (87, 92) | 76 (67, 85) |
| Hindfoot | 91 (88, 94) | 77 (70, 83) | 94 (92, 96) | 87 (80, 94) |
| Midfoot | 93 (90, 95) | 57 (44, 69) | 96 (95, 98) | 92 (88, 97) |
| Forefoot | 97 (96, 97) | 60 (54, 65) | 98 (98, 99) | 96 (96, 97) |
| The joints of 60 patients (47 patients with JIA and 13 controls) were assessed independently for joint inflammation on whole-body MRI scans by 3 readers. GAC2: Gwet’s agreement coefficient 2, G1: grade 1 synovitis, NA: negative specific agreement, OA: overall agreement, PA: positive specific agreement. Data are % (95% CI). | | | | |

**
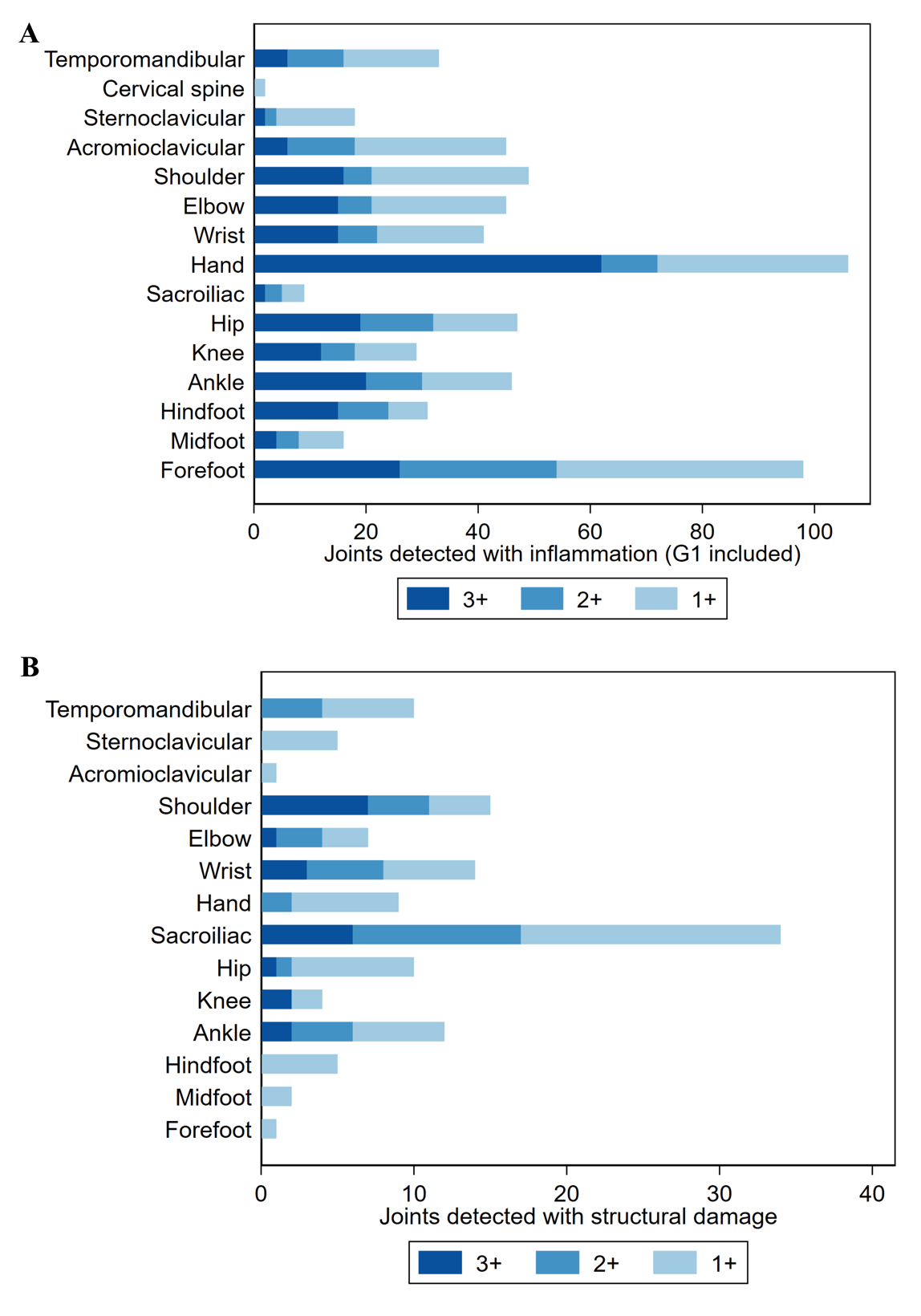
**

**Supplementary Figure S2.** Inter-reader agreement per joint assessed on whole-body MRI in 60 participants (JIA, controls) by 3 readers. A. For joint inflammation, defined as grade 1 (G1) or grade 2 synovitis in peripheral joints. B. For structural damage. Data presented as number of joints. Joints positive by three (3+), two (2+), and one (1+) reader(s) are shown in different colours.

**Supplementary Table S5.** The inter-reader OA, PA, NA, and GAC2 estimates for the identification of the same joint with structural damage

| **Joints** | **OA** | **PA** | | **NA** | **GAC2** |
| --- | --- | --- | --- | --- | --- |
| Temporomandibular | 94 (91, 96) | 29 (15, 47) | 97 (95, 98) | | 94 (90, 98) |
| Cervical spine | 100 (98, 100) | - | 100 (99, 100) | | - |
| Sternoclavicular | 97 (95, 98) | 0 (0, 28) | 99 (97, 99) | | 97 (95, 100) |
| Acromioclavicular | 99 (98, 100) | 0 (0, 66) | 100 (99, 100) | | 99 (98, 100) |
| Glenohumeral | 96 (93, 97) | 76 (64, 84) | 98 (96, 98) | | 95 (91, 99) |
| Elbow | 96 (94, 98) | 50 (31, 69) | 98 (97, 99) | | 96 (93, 99) |
| Wrist | 94 (91, 96) | 56 (42, 69) | 97 (95, 98) | | 93 (88, 97) |
| Hand | 100 (99, 100) | 18 (7, 39) | 100 (100, 100) | | 100 (99, 100) |
| Sacroiliac | 84 (80, 88) | 51 (42, 60) | 91 (88, 93) | | 78 (70, 87) |
| Hip | 95 (92, 97) | 31 (17, 50) | 97 (96, 98) | | 94 (91, 98) |
| Knee | 99 (97, 100) | 75 (51, 90) | 99 (99, 100) | | 99 (97, 100) |
| Ankle | 94 (92, 96) | 50 (35, 65) | 97 (95, 98) | | 94 (90, 98) |
| Hindfoot | 97 (95, 98) | 0 (0, 28) | 99 (97, 99) | | 97 (95, 100) |
| Midfoot | 99 (97, 100) | 0 (0, 49) | 99 (99, 100) | | 99 (97, 100) |
| Forefoot | 100 (100, 100) | 0 (0, 66) | 100 (100, 100) | | 100 (100, 100) |
| The joints of 60 patients (47 patients with JIA and 13 controls) were assessed independently for structural damage on whole-body MRI scans by 3 readers. GAC2: Gwet’s agreement coefficient 2, NA: negative specific agreement, OA: overall agreement, PA: positive specific agreement. Data are % (95% CI). | | | | | |


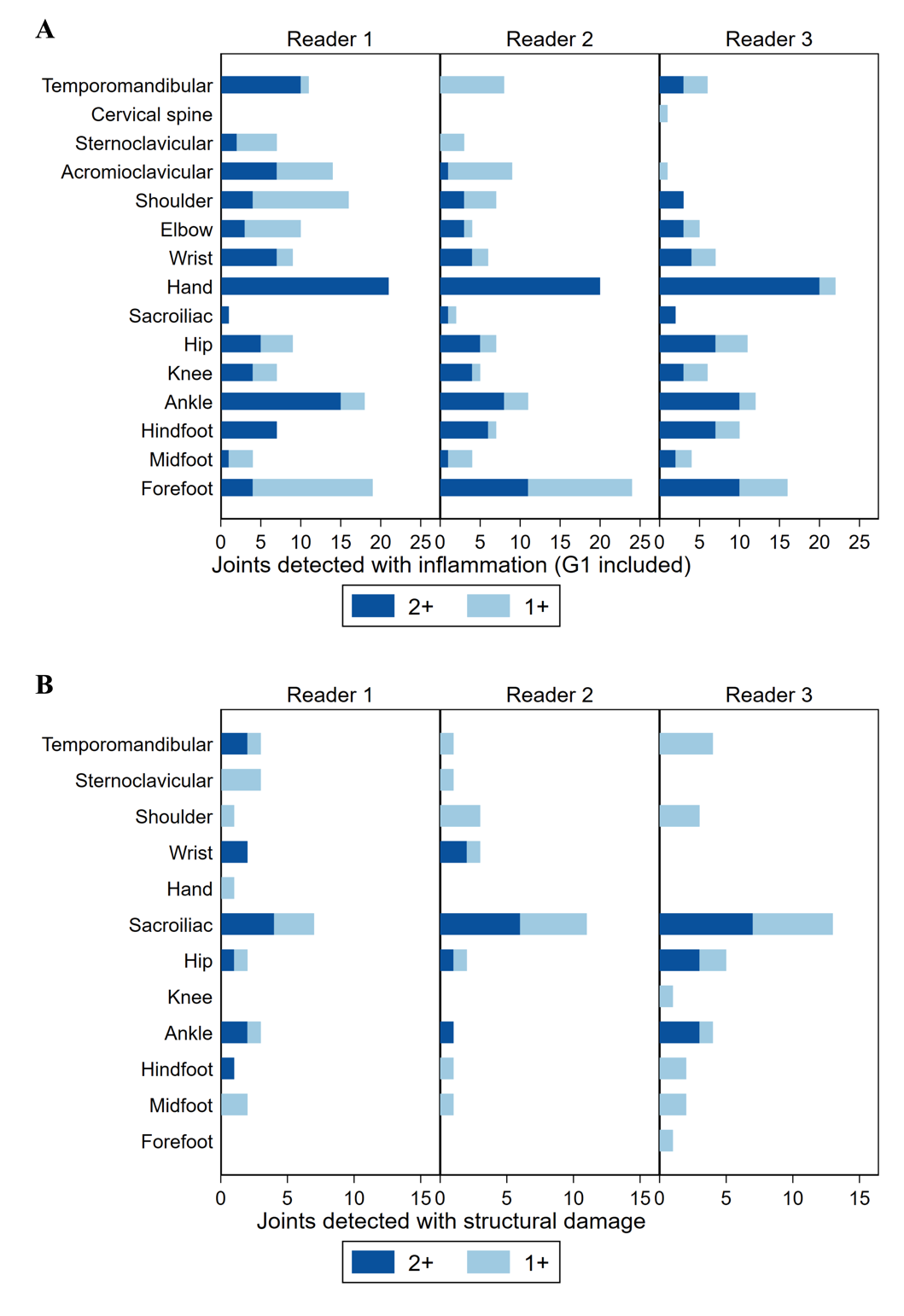


**Supplementary Figure S3.** Intra-reader agreement of readers 1, 2 and 3 on the presence of joint inflammation/structural damage per joint on the whole-body MRI scans of 20 (JIA, controls) participants. A. For joint inflammation, defined as grade 1 (G1) or grade 2 synovitis in peripheral joints. B. For structural damage. Data presented as number of joints, marked with different colours if the same joint was scored positive at both readings (2+), or at one reading (1+).

Supplementary Table S6. Inter-reader reliability on total WBMRI joints scores measured by ICC

| Readers combination | **Total joint inflammation score per patient (not including G1S)** | **Total joint inflammation score per patient (including G1S)** | **Total joint structural damage score per patient** |
| --- | --- | --- | --- |
| R1 vs R2 | 0.95 (0.92 – 0.97) | 0.95 (0.91 - 0.97) | 0.76 (0.63-0.85) |
| R2 vs R3 | 0.96 (0.92 – 0.98) | 0.97 (0.95 - 0.98) | 0.71 (0.54-0.82) |
| R1 vs R3 | 0.97 (0.95 – 0.98) | 0.96 (0.94 - 0.98) | 0.74 (0.61-0.84) |
| R1 vs R2 vs R3 | 0.96 (0.94 – 0.98) | 0.96 (0.94 - 0.98) | 0.74 (0.63-0.82) |
| Three readers assessed 81 joints/patient on whole-body MRI (WBMRI) for joint inflammation (grade 0-2, total joint inflammation score per patient: 0-162) and structural damage (0-1; total joint structural damage score per patient: 0-81). 4623 joints were scored in 60 patients (47 patients with JIA, 13 controls). ICCs were estimated for total joint inflammation score per patient (with and without including G1S) and for total joint structural damage scores; ICC form: 2-way random effects, absolute agreement, single rater. CI: confidence interval, ICC: intraclass correlation coefficient, G1S: grade 1 synovitis, G2S: grade 2 synovitis, R1: reader 1, R2: reader 2, R3: reader 3. | | | |

Supplementary Table S7. Intra-reader reliability on total WBMRI joint scores measured by ICC

| Readers | **Total joint inflammation score per patient (not including G1S)** | **Total joint inflammation score per patient (including G1S)** | **Total joint structural damage score per patient** |
| --- | --- | --- | --- |
| R1 | 0.98 (0.94-1.00) | 0.96 (0.91-0.98) | 0.71 (0.41-0.87) |
| R2 | 0.99 (0.97-1.00) | 0.98 (0.95-0.99) | 0.60 (0.22-0.82) |
| R3 | 0.99 (0.99-1.00) | 0.99 (0.97-1.00) | 0.71 (0.36-0.88) |
| Three readers assessed twice 81 joints/patient for joint inflammation (grade 0-2, total joint inflammation score per patient: 0-162) and structural damage (0-1; total joint structural damage score per patient: 0-81) on whole-body MRI (WBMRI). 1473 joints were scored in 20 patients (17 patients with JIA, 3 controls). ICCs were estimated for total joint inflammation score per patient (with and without including G1S) and for total joint structural damage scores; ICC form: 2-way mixed effects, absolute agreement, single rater. CI: confidence interval, ICC: intraclass correlation coefficient, G1S: grade 1 synovitis, G2S: grade 2 synovitis, R1: reader 1, R2: reader 2, R3: reader 3. | | | |
